# HCMV-miR-US33-5p promotes apoptosis of aortic vascular smooth muscle cells by targeting EPAS1/SLC3A2 pathway

**DOI:** 10.1186/s11658-022-00340-w

**Published:** 2022-05-20

**Authors:** Jian Dong, Shuangshuang Li, Zilin Lu, Pengcheng Du, Guangqin Liu, Mintao Li, Chao Ma, Jian Zhou, Junmin Bao

**Affiliations:** 1 Department of Vascular Surgery, Shanghai TCM-Integrated Hospital, Shanghai University of Traditional Chinese Medicine, Shanghai, China; 2 Department of Vascular Surgery, Changhai Hospital, Navy Medical University, Shanghai, China; 3 School of Health Science and Engineering, University of Shanghai for Science Technology, Shanghai, China

**Keywords:** Acute aortic dissection, Aortic vascular smooth muscle cells, Apoptosis, miRNAs, Endothelial PAS domain protein 1, SLC3A2

## Abstract

**Background:**

In patients with acute aortic dissection (AAD), increased vascular smooth muscle cell (VSMC) apoptosis has been found. Human cytomegalovirus (HCMV)-miR-US33-5p was significantly increased in the plasma of patients with AAD. However, the roles of miR-US33-5p in human aortic VSMC (HA-VSMC) apoptosis remain to be elucidated.

**Methods:**

In the current study, cell apoptosis was analyzed by flow cytometry, cell proliferation by CCK-8 assay, and differentially expressed genes by RNA sequencing. Luciferase reporter assay was used for binding analysis between miR-US33-5p and endothelial PAS domain protein 1 (EPAS1), and EPAS1 and amino acid transporter heavy chain, member 2 (SLC3A2). The enrichment degree of SLC3A2 promoter DNA was analyzed by chromatin immunoprecipitation assay. Quantitative reverse-transcription polymerase chain reaction (qRT-PCR) and immunoblotting were performed for measuring messenger RNA (mRNA) and protein levels, respectively.

**Results:**

It was found that HCMV infection inhibited proliferation but promoted HA-VSMC apoptosis by upregulating HCMV-miR-US33-5p. Transfection of HCMV-miR-US33-5p mimics the significant effect on several signaling pathways including integrin signaling as shown in the RNA sequencing data. Western blotting analysis confirmed that HCMV-miR-US33-5p mimics suppression of the activity of key factors of the integrin signal pathway including FAK, AKT, CAS, and Rac. Mechanistic study showed that HCMV-miR-US33-5p bound to the 3′-untranslated region of EPAS1 to suppress its expression, leading to suppression of SLC3A2 expression, which ultimately promoted cell apoptosis and inhibited cell proliferation. This was confirmed by the findings that silencing EPAS1 significantly reduced the SLC3A2 expression and inhibited proliferation and key factors of integrin signal pathway.

**Conclusions:**

HCMV-miR-US33-5p suppressed proliferation, key factors of integrin signal pathway, and EPAS1/SLC3A2 expression, but promoted HA-VSMC apoptosis. These findings highlighted the importance of HCMV-miR-US33-5p/EPAS1/SCL3A2 signaling and may provide new insights into therapeutic strategies for AAD.

**Supplementary Information:**

The online version contains supplementary material available at 10.1186/s11658-022-00340-w.

## Background

Acute aortic dissection (AAD) is an emergency disease with high mortality [1], and hypertension is a common risk factor [2]. Its diagnosis mainly relies on computed tomography (CT) and magnetic resonance angiography (MRA) [3]. Although sensitivity (98–100%) and specificity (95–98%) are high, community hospitals remain reluctant to use CT and MRA due to the inconvenience of these methods [4]. In addition, a fraction of patients do not show typical clinical manifestations, and CT and MRA can in these cases lead to misdiagnosis or no diagnosis at all [5, 6].

Human cytomegalovirus (HCMV), a member of the Herpesviridae family, may be the most ubiquitous of human infections [7–9]. HCMV infection is closely related to cardiovascular diseases, as suggested by epidemiological studies [10, 11]. HCMV encodes at least 26 microRNAs (miRNAs), scattered in the viral genome in the form of a single miRNA or gene cluster [12, 13]. HCMV-encoded miRNAs have been found in plasma. Plasma miRNA has been reported as a biomarker for disease diagnosis and treatment [14]. Increasing evidence has shown that miRNA is not only involved in the physiological process of cardiovascular development but also plays a crucial role in the pathological process of cardiovascular disease [15]. Our previous study showed that HCMV-miR-US33-5p plasma levels were significantly higher in patients with AAD than in healthy individuals or patients with non-AAD chest pain [16]. HCMV-miR-US33-5p targets the 3′-untranslated region (UTR) of HCMV US29 and host STX3, inhibiting viral DNA replication by suppressing US29 and STX3 expression, and promoting HCMV latent infection [17]. However, the functions of HCMV-miR-US33-5p in AAD are unknown.

Many studies have revealed that increased VSMC apoptosis is involved in the pathogenesis of AAD [18–20]. Endothelial PAS domain protein 1 (EPAS1), a member of the basic-helix-loop-helix/PAS domain-containing transcription factors [21], is selectively expressed in endothelial cells and VSMCs [21–23]. EPAS1 has been shown to be related to cardiovascular disease including cardiac hypertrophy [24, 25] and is also closely related to apoptosis, as indicated in studies. For example, EPAS1 potentiates Fas-mediated chondrocyte apoptosis [26]. Silencing EPAS1 inhibited cell proliferation and induced cell apoptosis of pancreatic cancer cells [27]. Studies showed that amino acid transporter heavy chain, member 2 (SLC3A2) is involved in cell growth, proliferation, and survival [28]. SLC3A2 functioned as an important factor in endoplasmic reticulum stress, and inhibiting SLC3A2 enhanced cardiomyocyte apoptosis, as reported by Liu et al. [29]. SLC3A2 deficiency led to a significant reduction of VSMC proliferation in the neointima [30, 31].

Regardless of advances in miR-US33-5p, EPAS1, and SLC3A2 studies, their roles in human aortic VSMC (HA-VSMC) apoptosis remain to be further elucidated. Here, the effect of HCMV-miR-US33-5p on HA-VSMC proliferation and apoptosis was studied, and how EPAS1 and SLC3A2 contribute to HCMV-miR-US33-5p-induced apoptosis of HA-VSMCs was explored.

## Materials and methods

### Cell and virus culture

HA-VSMCs and human embryonic lung fibroblasts (HELFs) were obtained from ATCC (Manassas, VA, USA) and cultured with Dulbecco’s modified Eagle’s medium (DMEM) with 10% fetal bovine serum (FBS), 4-(2-hydroxyethyl)-1-piperazineethanesulfonic acid (HEPES), l-glutamine, and pyridoxine HCl (Sigma-Aldrich, MO, USA), while HEK293 cells were obtained from the Chinese Academy of Sciences cell bank and cultured with DMEM with 10% FBS. The AD169 strain of human cytomegalovirus (HCMV) and AD169 (ΔUS33) with the *US33* gene knocked out were generated using TB40-BAC_KL7_-UL32EGFP (gifted by Dr. Katharina Gohring, Tubingen University, Germany) by Dr. Gan from Anhui Medical University, Department of Microbiology [32]. The virus was propagated in HELF cells and stored in liquid nitrogen.

### Ultraviolet (UV) inactivation of viruses

HCMV AD169 and AD169 (ΔUS33) were grown in HELF cells and purified by ultracentrifugation on a 10–50% sucrose gradient according to Zucker’s method [33]. The virus was titrated by plaque-forming assay using HELF cells and stored in small aliquots at −80 °C. Purified HCMV was irradiated with 5 × 10^3^ µJ/m^2^ using a UV crosslinker (Agilent Technologies, CA, USA). Under these conditions, no infectious virions were detected.

### Cell transfection

The following were all purchased from GenePharma (Shanghai, China): HCMV-miR-US33-5p mimics (mimics sequence: GAUUGUGCCCGGACCGUGGGCG), control miRNA (UCACCGGGUGUAAAUCAGCUUG), EPAS1, and SLC3A2 small interfering RNAs and their corresponding controls (Table 1). Transfection was carried out in accordance with the Lipofectamine 3000 instructions (Invitrogene, CA, USA). The miRNA or siRNA and Lipofectamine 3000 were diluted with Opti-MEM ((Invitrogene) and incubated for 5 min. After mixing the two in proportion, they were incubated for 20 min to form an RNA/Lipofectamine 3000 complex. The complex was added to the cell culture medium, and the cells were harvested after 24-h incubation.

**Table 1:** Small interfering RNAs

Name	Sequence (5′–3′)
siEPAS1-1	CCAAGAGUCACCAGAACUUUU
siEPAS1-2	CCUGAAGAUUGAAGUGAUUUU
siSLC3A2-1	GCCAGGCUGAUAUUGAAUUUU
siSLC3A2-2	GGUUCGGGACAUAGAGAAUUU
siNC	CAGUACUUUUGUGUAGUACAA

### Cell viability assay

Cell viability was measured with CCK-8 (Beyotime Biotechnology, C0037, Shanghai). Each group’s cells were trypsinized and then inoculated at 2000 cells/well in 96-well plates. At 0, 24, 48, and 72 h after cell inoculation, 10 μl CCK-8 solution was loaded and incubated for 1 h. The optical density at 450 nm (OD450) was recorded using a microplate reader.

### Apoptosis detection

Apoptosis was measured with the Annexin V-PI analysis kit (Beyotime, C1062) on a flow cytometer (FACScan; BD). In short, the cells were harvested, washed, resuspended, mixed with Annexin V-fluorescein isothiocyanate (FITC) and propidium iodide (PI), incubated in the dark for 20 min, and analyzed in a flow cytometer (BD Biosciences, NJ, USA).

### Caspase-3 activity

Caspase-3 activity was measured with the GreenNuc caspase-3 assay kit for live cells (Beyotime, C1168) by following the manufacturer’s instructions. After treatment, the cells were collected, washed, and mixed with GreenNuc caspase-3 substrate. After 30-min incubation at 37 °C in the dark, the caspase-3 activity levels were determined by a fluorescence microplate reader. The relative caspase-3 activity was calculated according to the treated-to-control cell ratio.

### RNA sequencing

Total RNA was isolated with TRIzol (Invitrogen). mRNA was purified with the Dynabeads mRNA purification kit (Invitrogen) and fragmented to construct the complementary DNA (cDNA) library sequenced by Illumina HiSeq 2000 (Illumina, CA, USA). Adapter sequences and low-quality (> 50% of base pairs where [QA] < 15) data were removed. Data were annotated using Tophat software (version 2.0.13), and for combinatorial evaluation of transcriptional data and differential expression detection (|log _twofold_ change (FC)|> 1.0, false discovery rate (FDR) < 0.05), Cufflinks (version 2.2.1) was used. Heatmap was drawn using R software. For pathway analysis, Gene Ontology (GO) and Kyoto Encyclopedia of Genes and Genomes (KEGG) were used. Data analysis was performed using the Majorbio Cloud Platform (www.majorbio.com) [34].

All original sequence data have been deposited in NCBI’s Sequence Read Archive database (http://www.ncbi.nlm.nih.gov/sra, AC: SUB11206773).

### Fluorescence quantitative PCR

Total RNA was extracted with TRIzol (Invitrogen). Then, for miR-US33-5p detection, the Hairpin-it microRNA RT-PCR quantitation kit (GenePharma) was used. The PCR reaction conditions were 95 °C, 10 min; 40 × (95 °C, 12 s, 62 °C, 40 s). After reverse transcription using the MMLV reverse transcriptase kit (Takara, Dalian, China), quantitative detection of EPAS1 and SLC3A2 mRNA was performed using the SYBR Premix Ex Taq kit (Takara) with the following conditions: 50 °C, 2 min, 95 °C, 10 min; 40 × (95 °C, 20 s, 60 °C, 30 s, 72 °C, 30 s). All PCR reactions were performed on the ABI StepOne Plus system (Applied Biosystem, CA, USA). Relative expression of miR-US33-5p and target genes was calculated by the 2^−ΔΔCT^ method with RNU6-1 and glyceraldehyde 3-phosphate dehydrogenase (GAPDH) as internal control, respectively. Table 2 presents the primers obtained from Genechem (Shanghai, China).

**Table 2:** Primer sequences

Name	Accession number	Sequences (5′–3′)
HCMV-miR-US33-5p	MIMAT0001584	F: GGATTGTGCCCGGACCG R: AGTGCAGGGTCCGAGGTATT
		RT 5'GTCGTATCCAGTGCAGGGTCCGAGGTATTCGCACTGGATACGACCGCCCA3'
RNU6-1	NR_004394.1	F: CTCGCTTCGGCAGCACA R: AACGCTTCACGAATTTGCGT
EPAS1	NM_001430.5	F: AACGCCAGCACACTATTTAC R: AGAACAGACATGCACCAAAC
SLC3A2	NM_001012662.3	F: TACCCTCTAACCCTGTTC R: CCCGTTTCTACTGTAACC
GAPDH	NM_001256799.2	F: AATCCCATCACCATCTTC R: AGGCTGTTGTCATACTTC

### Immunoblotting

Proteins were extracted with radioimmunoprecipitation buffer containing proteinase inhibitor (Beyotime), and then separated by sodium dodecyl sulfate (SDS)-polyacrylamide gel electrophoresis (PAGE) (30 μg per sample) before nitrocellulose membrane transfer (Millipore Biotech, MA, USA). The membranes were blocked with 5% skim milk and probed with primary and horseradish peroxidase (HRP)-conjugated rabbit secondary antibodies (Beyotime). To detect the signal, an enhanced chemiluminescence system (ECL, Millipore Biotech) was used. Band density was quantified using ImageJ software (http://rsb.info.nih.gov/ij/, MD, USA) and normalized to GAPDH. The primary antibodies were as follows: EPAS1 (Abcam, ab222396, 1:2000), SLC3A2 (Abcam, ab244356, 1:1000), p-AKT (CST, #4060, 1:1000), AKT (CST, #4685, 1:1000), p Y576-FAK (Abcam, ab76120, 1:1000), FAK (Abcam, ab40794, 1:500), Cas (Abcam, ab92514, 1:1000), pY20-Cas (BioSource International, AHO0681, 1:1000), cleaved caspase-3 (CST, #9661, 1:1000), and GAPDH (CST, #5174, 1:1000).

### GTP-Rac assay

GTP-Rac quantitative analysis in different cell groups was carried out with the GTP-Rac detection kit (CST, #8815) as described previously [35]. Cells were lysed with a lysis buffer, and then the lysates were incubated in binding buffer containing 20 μg GST-p21-activated kinase binding domain and glutathione–agarose beads for 3 h at 4 °C. The beads were washed and eluted. Total Rac and bound Rac (GTP-Rac) were analyzed by immunoblotting using anti-Rac antibody (CST, #4651, 1:1000).

### Dual luciferase reporter gene analysis

Wild-type and mutant EPAS1 3′-UTR plasmids, wild-type (WT)-pGL3-EPAS1, and mutant-pGL3-EPAS1 plasmids were created by GenePharma to analyze the miR-US33-5p and EPAS1 3′-UTR interaction. The luciferase reporter gene plasmid, internal reference plasmid, and miR-US33-5p were cotransfected into HEK293 cells Lipofectamine 3000. WT pGL3-Basic-SLC3A2 (ACGTGC) plasmid and the mutant pGL3-Basic-SLC3A2 (TAAAAA) plasmid were created by GenePharma to analyze the EPAS1 regulation on SLC3A2 promoter. The plasmid pex-3-EPAS1 expressing EPAS1 was cotransfected with pGL3-Basic-SLC3A2 (ACGTGC), pGL3-Basic-SLC3A2 (TAAAAA) plasmid, and the control plasmid pGL3-Basic into HEK293 cells. After 48 h, the cells were lysed, and luminescence was measured on a luminometer. The results were normalized using renin fluorescence as internal reference.

### RNA binding protein immunoprecipitation (RIP) assay

The RIP experiment was performed according to the EZMagna RIP kit’s instructions (Millipore). HCMV was used to infect HA-VSMCs at multiplicity of infection (MOI) of 5. After 72 h, the cells were lysed with RIP lysis buffer and incubated with magnetic beads bound to anti-Ago2 antibody (CST, 2897), or normal mouse immunoglobulin G (IgG). Proteins were removed by proteinase K at 55 °C for 30 min. RNA purity and content were evaluated by NanoDrop 1000 (Thermo Fisher Scientific, IL, USA). RNA was purified using RNeasy Micro kit (Qiagen, Germany, Dusseldorf) and detected by fluorescence quantitative PCR.

### Chromatin immunoprecipitation (ChIP)-qPCR assay

For ChIP analysis, SimpleChIP Plus sonication chromatin IP kit (CST, #9005) was used as described previously [36]. After the HCMV-infected HA-VSMCs were cross-linked with formaldehyde, the cells were sonicated for chromatin fragmentation, and then incubated overnight with anti-human EPAS1 antibody (CST, #59973) at 4 °C. Then, Protein G magnetic beads (CST, #9006) were added and incubated with shaking for 2 h at 4 °C. DNA on the beads was washed and eluted. A standard curve with 2% input chromatin DNA in serial dilutions (undiluted, 1:5, 1:25, and 1:125) was generated and used for measuring the enrichment degree of the SLC3A2 promoter DNA. PCR products were subjected to agarose gel electrophoresis.

### Statistical analysis

Statistical analysis was done by SPSS version 17.0 (SPSS, Chicago, IL, USA), and data are expressed as mean ± standard deviation (SD). Differences between two groups and among more than two groups were measured by Student’s *t*-test and one-way analysis of variance (ANOVA), respectively. *P* < 0.05 was defined as statistically significant.

## Results

### HCMV promotes HA-VSMC apoptosis by upregulating HCMV-miR-US33-5p

**Fig. 1:**
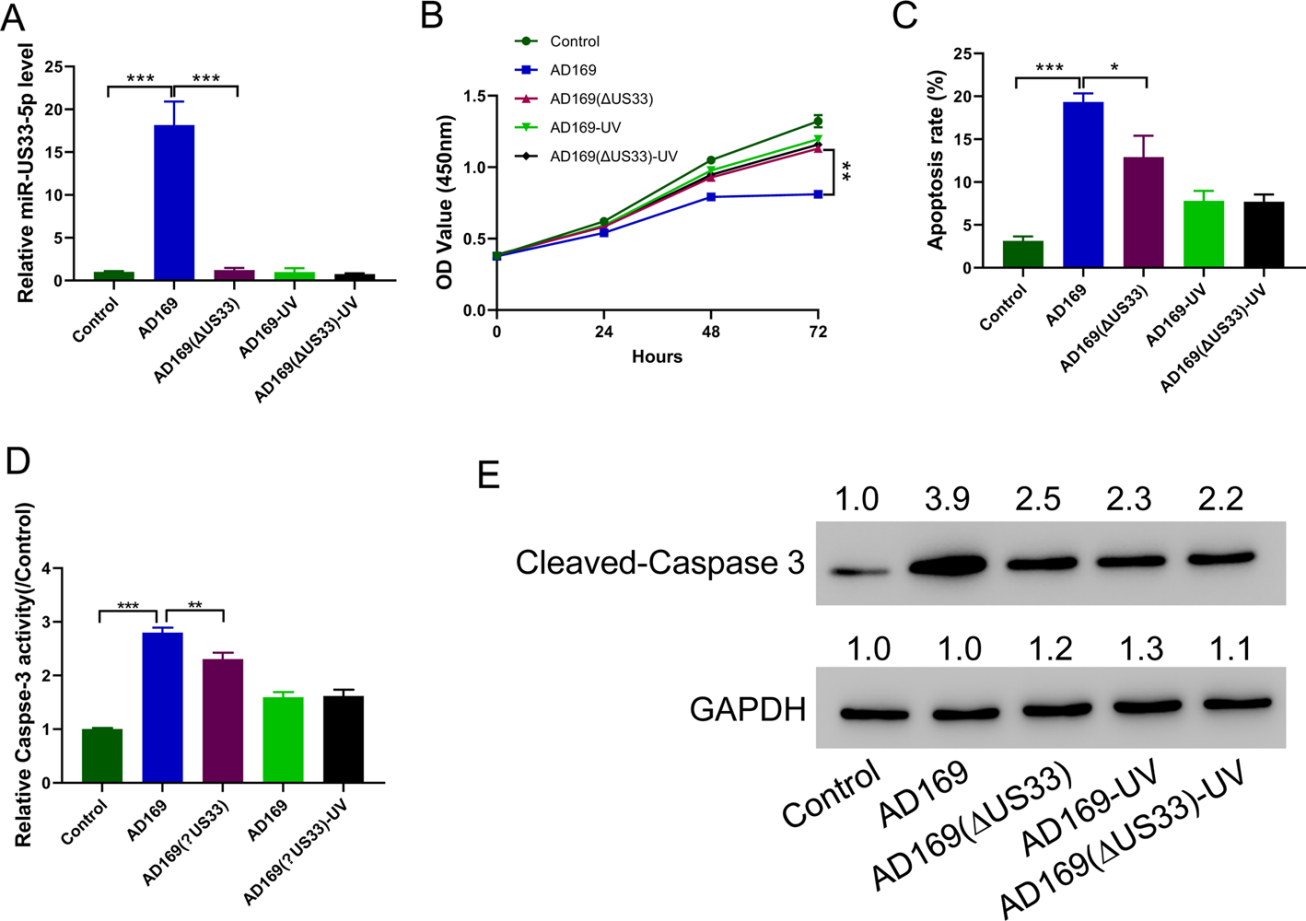
HCMV promotes apoptosis by upregulating HCMV-miR-US33-5p. **A** qRT-PCR analysis of HCMV-miR-US33-5p expression level in HA-VSMCs infected with different viruses. **B** CCK-8 assay showed that HCMV AD169 virus infection inhibited HA-VSMC proliferation, while ultraviolet inactivation of the virus (UV) or the AD169 (ΔUS33) virus showed a greatly reduced inhibitory effect on HA-VSMC proliferation. **C**–**E** Flow cytometry assay (**C**), caspase-3 activity assay (**D**), and Western blot analysis of cleaved caspase-3 (**E**) indicated that HCMV AD169 virus infection promoted HA-VSMC apoptosis, while UV-inactivated virus (UV) or AD169 (ΔUS33) virus showed a greatly reduced stimulatory effect on HA-VSMC apoptosis. Western blot was performed three times; representative images are presented. Numbers above lanes indicate the relative protein level, normalized relative to control samples. *P < 0.05, **P < 0.01, ***P < 0.001 (*n* = 3, ANOVA analysis)

To explore the role of HCMV in HA-VSMC proliferation and apoptosis, HA-VSMCs were infected with HCMV AD169 virus, AD169 (ΔUS33), UV-inactivated AD169 (AD169-UV), and AD169 (ΔUS33)-UV. HCMV AD169 virus infection significantly increased miR-US33-5p levels, while AD169 with the *US33* gene knocked out (ΔUS33) and UV-inactivated AD169-UV did not affect the miR-US33-5p level in HA-VSMCs (Fig. 1A). CCK-8 assay results showed that HCMV AD169 virus infection inhibited HA-VSMCs proliferation, while UV-inactivated virus (UV) or AD169 (ΔUS33) virus showed a greatly reduced inhibitory effect on HA-VSMC proliferation (Fig. 1B). Then, flow cytometry analysis (Fig. 1C), caspase-3 activity assay (Fig. 1D), and Western blotting of cleaved caspase-3 (Fig. 1E) were performed to determine cell apoptosis. HCMV AD169 virus infection significantly promoted HA-VSMC apoptosis, while UV-inactivated virus (UV) or AD169 (ΔUS33) virus showed a greatly reduced stimulatory effect on promoting HA-VSMCs apoptosis. Together, these results suggest that HCMV promotes HA-VSMC apoptosis by upregulating miR-US33-5p.

### HCMV-miR-US33-5p inhibits proliferation of HA-VSMCs and promotes their apoptosis

**Fig. 2:**
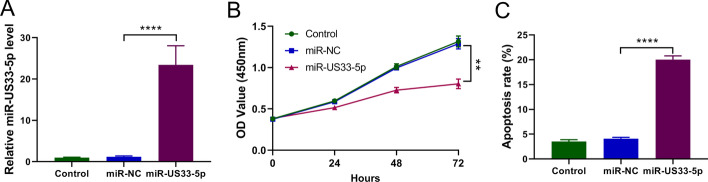
HCMV-miR-US33-5p inhibits HA-VSMC proliferation and promotes their apoptosis. HA-VSMCs were transfected with HCMV-miR-US33-5p. **A** qRT-PCR analysis of HCMV-miR-US33-5p expression level. **B** CCK-8 assay indicated that HCMV-miR-US33-5p significantly inhibited cell proliferation starting from 24 h after transfection. **C** Flow cytometry analysis indicated that HCMV-miR-US33-5p significantly increased HA-VSMC apoptosis. *P < 0.05, **P < 0.01, ***P < 0.001 (*n* = 3, ANOVA analysis)

To further explore the effect of HCMV-miR-US33-5p on HA-VSMCs, HCMV-miR-US33-5p was transfected into HA-VSMCs (Fig. 2A). Transfection of HCMV-miR-US33-5p significantly inhibited cell proliferation starting from 24 h after transfection (Fig. 1B), and transfection of HCMV-miR-US33-5p also promoted HA-VSMC apoptosis (Fig. 1C). These findings suggest that HCMV-miR-US33-5p inhibits HA-VSMC proliferation and promotes HA-VSMC apoptosis.

### Identification of differentially expressed genes (DEGs) following HCMV-miR-US33-5p transfection by RNA-seq analysis

**Fig. 3:**
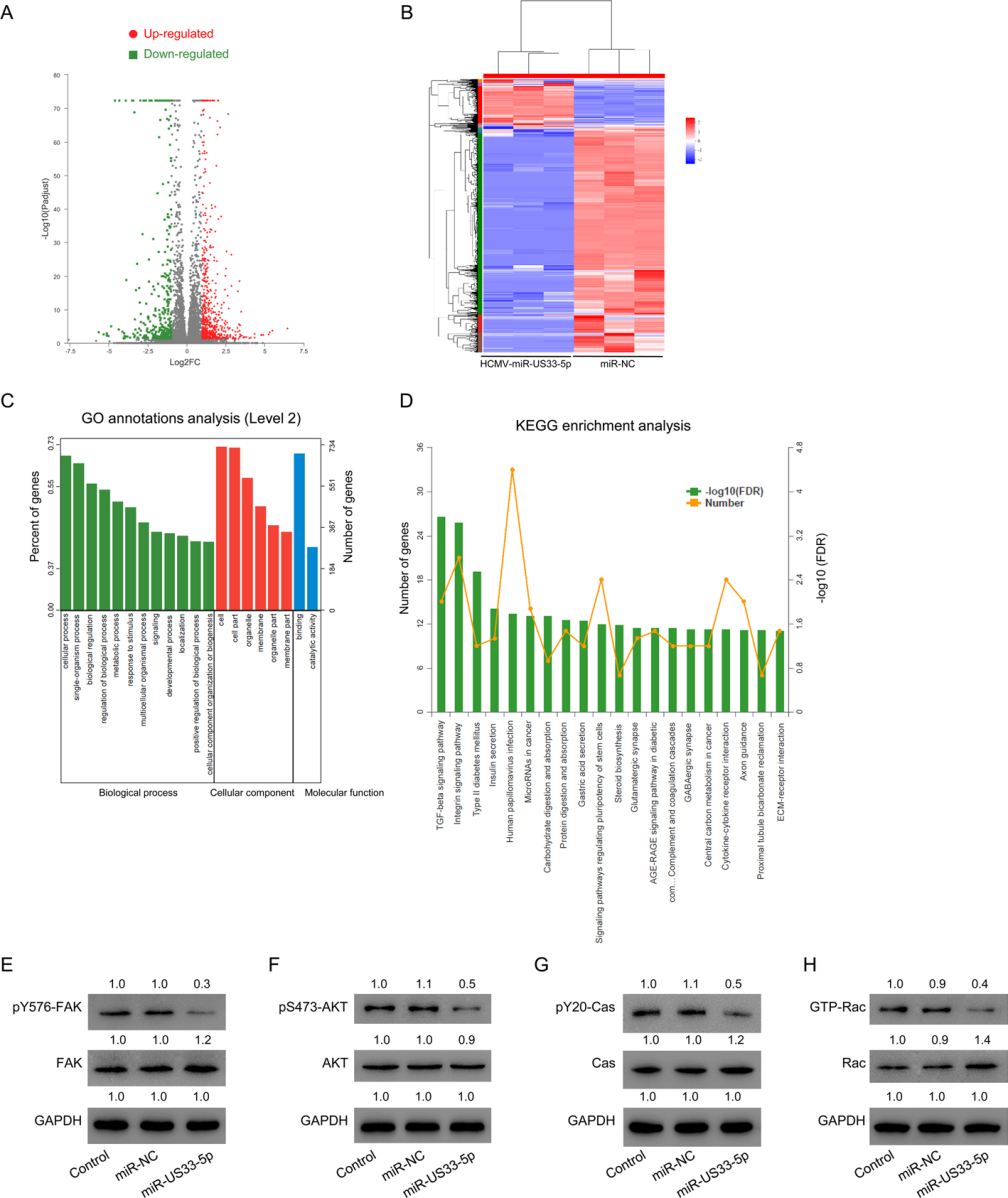
HCMV-miR-US33-5p inhibits key factors of integrin signaling pathway in HA-VSMCs. **A** Volcano plot for DEGs in the control (*n* = 3) and HCMV-miR-US33-5p transfection (*n* = 3) groups. Green and red points represent genes downregulated and upregulated in the HCMV-miR-US33-5p transfection group, respectively. **B** Heatmap for DEGs in the control (*n* = 3) and HCMV-miR-US33-5p transfection (*n* = 3) groups. **C** GO annotations; **D** KEGG enrichment analysis. **E**–**H** Western blot analysis of p-FAK (**E**), p-AKT (**F**), pY20 CAS (**G**), and GTP-Rac (**H**) in HA-VSMCs. Western blot was performed three times; representative images are presented. Numbers above lanes indicate the relative protein level, normalized relative to the control samples

To investigate how transfection of HCMV-miR-US33-5p affects HA-VSMC proliferation and apoptosis, high-throughput sequencing was used to find DEGs in HA-VSMCs transfected with HCMV-miR-US33-5p mimics or miR-NC. A total of 1003 DEGs were identified, of which 401 were downregulated and 602 were upregulated (Fig. 3A; Additional file 2: Table S1, Additional file 3: Table S2) using a cutoff of |log_2_ FC| > 1.0, FDR < 0.05. GO and KEGG analysis of these DEGs showed that transforming growth factor (TGF)-β signaling, integrin signaling, and type II diabetes mellitus signaling pathway were the signaling pathways most affected by transfection of HCMV-miR-US33-5p mimics (Fig. 3C, D). The integrin signaling pathway, which plays critical roles in cell apoptosis and AAD, attracted our attention. Thus, its key factors were analyzed. The Western blot results also showed that transfection of HCMV-miR-US33-5p mimics decreased the phosphorylation of key factors of the integrin signal pathway, including FAK, AKT, and CAS (Fig. 3E–G). Rac activation was also suppressed by HCMV-miR-US33-5p mimics (Fig. 3H). Together, these data suggest that HCMV-miR-US33-5p might suppress integrin signaling in HA-VSMCs.

### HCMV-miR-US33-5p downregulates SLC3A2 in HA-VSMCs, and silencing of SLC3A2 promotes cell apoptosis

**Fig. 4:**
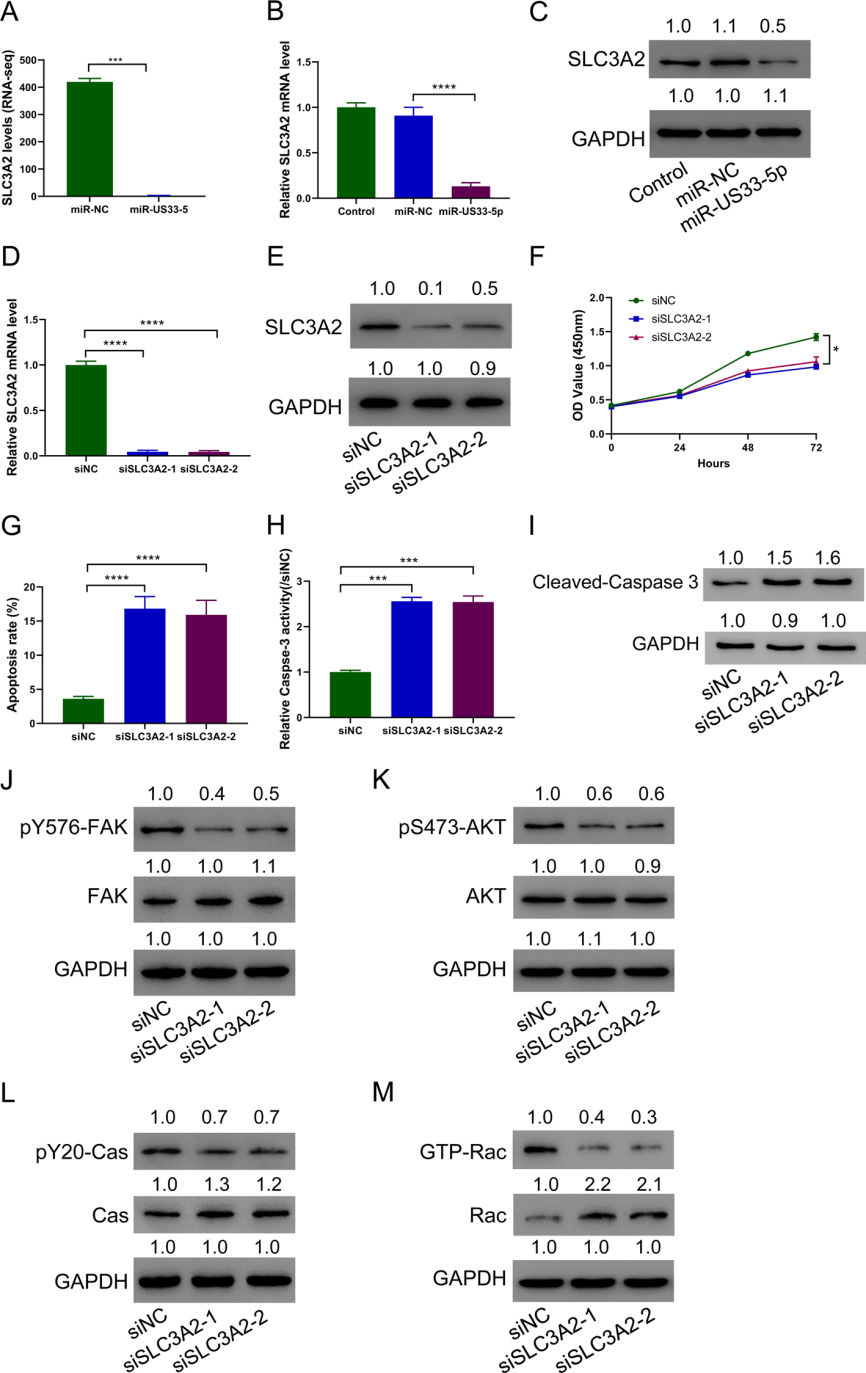
Downregulating SLC3A2 promotes HA-VSMC cell apoptosis. HCMV-miR-US33-5p was transfected into HA-VSMCs. **A** RNA sequencing data showing differential expression of SLC3A2 between HCMV-miR-US33-5p and control groups. **B**, **C** SLC3A2 expression in HA-VSMCs. **D**, **E** SLC3A2 expression in HA-VSMCs after silencing SLC3A2. **F** CCK-8 assay showed that transfection of siRNA targeting SLC3A2 inhibited HA-VSMC proliferation. **G**–**I** Flow cytometry assay (**G**), caspase-3 activity assay (**H**), and Western blot analysis of cleaved caspase-3 (**I**) showed that transfection of siRNA targeting SLC3A2 promoted HA-VSMC apoptosis. **J**–**M** Western blot analysis of the effect of SLC3A2 siRNA on p-FAK (**J**), p-Akt (**K**), pY20CAS (**L**), and GTP-Rac (**M**) in HA-VSMCs. Western blot was performed three times; representative images are presented. Numbers above lanes indicate the relative protein level, normalized relative to control samples. *P < 0.05, ***P < 0.001, ****P < 0.0001 (*n* = 3, ANOVA analysis)

High-throughput sequencing found that SLC3A2 decreased significantly after transfection of HCMV-miR-US33-5p mimics (Fig. 4A). This result was verified by PCR and immunoblots (Fig. 4B, C). To analyze the role of SLC3A2, SLC3A2 was silenced in HA-VSMCs (Fig. 4D, E). Silencing SLC3A2 significantly decreased the proliferation ability of HA-VSMCs (Fig. 4F), but increased their apoptosis level (Fig. 4G), relative caspase-3 activity (Fig. 4H), and cleaved caspase-3 expression (Fig. 4I). SLC3A2 silencing also decreased key factors of the integrin signal pathway including p-FAK, p-AKT, p-CAS, and GTP-Rac (Fig. 4J–M). These findings demonstrate that HCMV-miR-US33-5p downregulates SLC3A2 in HA-VSMCs, and SLC3A2 silencing promotes cell apoptosis.

### HCMV-miR-US33-5p downregulates SLC3A2 by targeting EPAS1

**Fig. 5:**
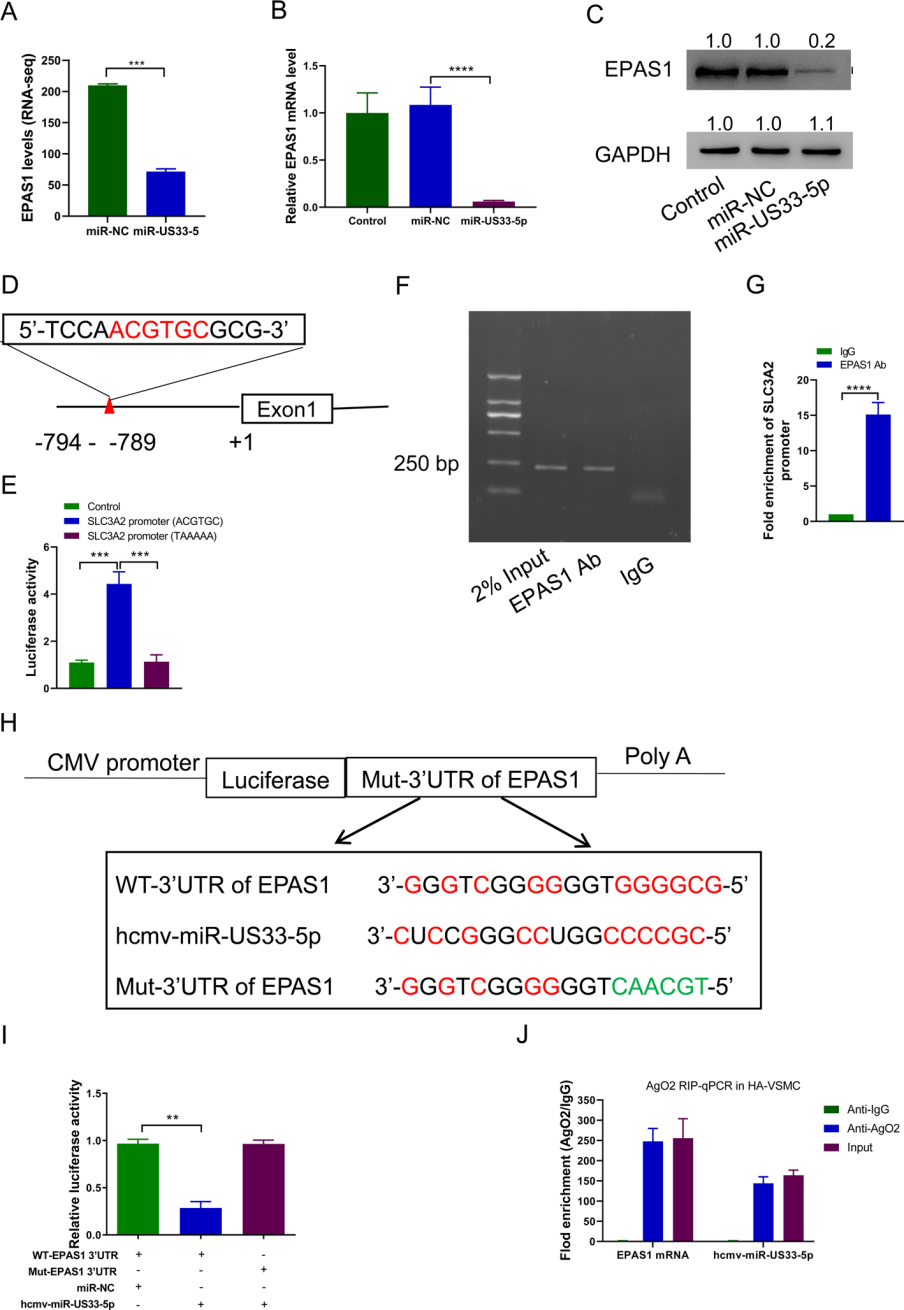
HCMV-miR-US33-5p downregulates SLC3A2 by targeting EPAS1. **A** RNA sequencing data showing EPAS1 differential expression between the HCMV-miR-US33-5p and control groups. **B**, **C** HCMV-miR-US33-5p was transfected into HA-VSMCs. qRT-PCR (**B**) and Western blot (**C**) were used to analyze the EPAS1 expression level in HA-VSMCs. Western blot was performed three times; representative images are presented. Numbers above lanes represent the relative protein level, normalized relative to control samples. **D** Potential EPAS1 binding sites in SLC3A2 promoter region. **E** Luciferase reporter assay of the effect of EPAS1 on the SLC3A2 promoter. **F** ChIP assay showed that EPAS1 bound to SLC3A2 promoter. **G** ChIP-qPCR assay of the enrichment of EPAS1 to SLC3A2 promoter. **H** Potential HCMV-miR-US33-5p binding site in EPAS1 promoter. **I** Luciferase reporter assay of the effect of HCMV-miR-US33-5p on the EPAS1 promoter. **J** RIP analysis of the enrichment of Ago2 on 3′-UTR of EPAS1 and HCMV-miR-US33-5p. **P < 0.01, ***P < 0.001 (*n* = 3, ANOVA analysis for **A** and **D**; Student’s *t*-test for **F**)

High-throughput sequencing data analysis found that EPAS1 decreased significantly after transfection of HCMV-miR-US33-5p mimics (Fig. 5A), which was verified by fluorescence quantitative PCR and Western blot (Fig. 5B, C). The SLC3A2 promoter sequence was then analyzed, and a typical EPAS1 binding site sequence at positions −789 to −794 (Fig. 5D) was found. So, the interaction between EPAS1 and SLC3A2 was tested. Luciferase reporter assay showed that EPAS1 promoted upregulation of wild-type SLC3A2 promoter (ACGTGC) activity but showed no effects on mutant SLC3A2 promoter (TAAAAA) (Fig. 5E). ChIP-qPCR indicated that EPAS1 was significantly enriched in the SLC3A2 promoter region (Fig. 5F, G), indicating that EPAS1 bound to the SLC3A2 promoter. Bioinformatics predictions indicated that there are HCMV-miR-US33-5p binding sites in the 3′-UTR region of EPAS1 (Fig. 5H). So, the interaction between EPAS1 and HCMV-miR-US33-5p was tested. The luciferase reporter assay results indicated that HCMV-miR-US33-5p bound to 3′-UTR of EPAS1 to inhibit its expression (Fig. 5I). RIP test showed that that Ago2 antibody simultaneously enriched EPAS1 mRNA and HCMV-miR-US33-5p, indicating that the two can form RNA-induced silencing complex (Fig. 5J). These data indicate that HCMV-miR-US33-5p downregulates SLC3A2 by targeting EPAS1.

### Downregulating EPAS1 in HA-VSMCs promotes cell apoptosis

**Fig. 6:**
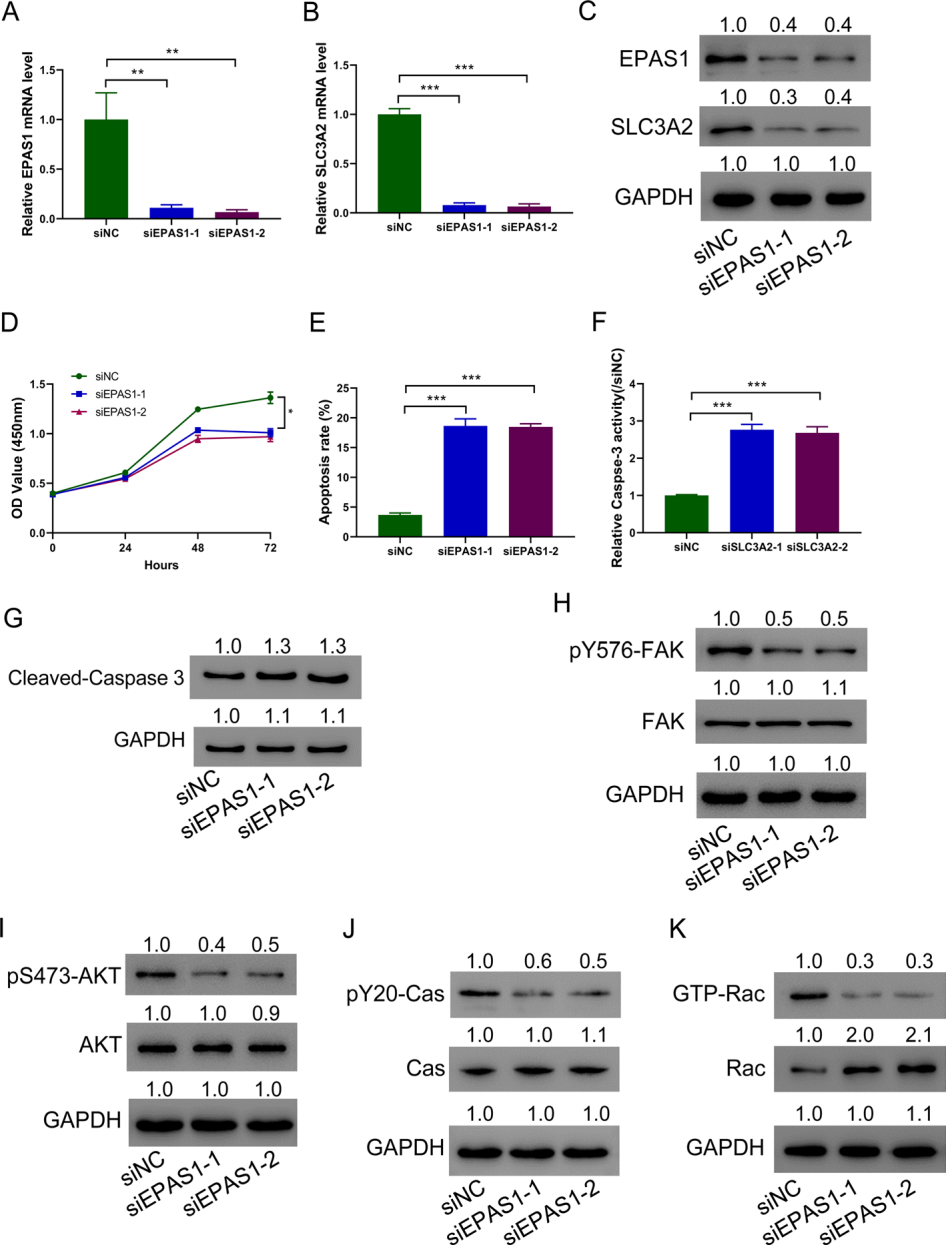
Downregulating EPAS1 in HA-VSMCs promotes cell apoptosis. **A**–**C** EPAS1 was silenced in HA-VSMCs. qRT-PCR (**A**) and immunoblotting (**C**) analysis of EPAS1 in HA-VSMCs. qRT-PCR (**B**) and immunoblotting (**C**) analysis of SLC3A2 in HA-VSMCs. **D** Silencing SLC3A2 inhibited HA-VSMC proliferation. **E**, **G** Flow cytometry assay (**E**), caspase-3 activity assay (**F**), and Western blot analysis of cleaved caspase-3 (**G**) showed that silencing SLC3A2 promoted HA-VSMC apoptosis. **H**–**K** p-FAK (**H**), p-AKT (**I**), pY20CAS (**J**), and GTP-Rac (**K**) immunoblotting in HA-VSMCs. Western blot was performed three times; representative images are presented. Numbers above lanes indicate the relative protein level, normalized relative to control samples. ***P < 0.001, ****P < 0.0001 (*n* = 3, ANOVA analysis)

To further analyze the effects of EPAS1, EPAS1 was successfully silenced in A-VSMCs (Fig. 6A). Silencing EPAS1 significantly reduced the SLC3A2 expression (Fig. 6B). Western blots further confirmed the above-mentioned results (Fig. 6C). It was found that silencing EPAS1 in HA-VSMCs significantly inhibited the proliferation (Fig. 6D) but promoted apoptosis (Fig. 6E), relative caspase-3 activity (Fig. 6F), and cleaved caspase-3 expression (Fig. 6G) of HA-VSMCs. At the same time, silencing EPAS1 also caused integrin signal pathway suppression as shown by decreased p-FAK, p-AKT, p-CAS, and GTP-Rac (Fig. 6H–K). These data demonstrate that silencing EPAS1 promotes HA-VSMC apoptosis.

## Discussion

HCMV is closely associated with various cardiovascular diseases, including hypertension, atherosclerosis, and graft vascular disease [37, 38]. Here, it is reported that HCMV promotes HA-VSMC apoptosis, which is correlated with the pathogenesis of AAD [18–20], by upregulating an HCMV-encoded miRNA, HCMV-miR-US33-5p. It was also proved that HCMV-miR-US33-5p inhibited the key factors of the integrin signaling pathway in HA-VSMCs. Further studies indicated that miR-US33-5p targeted EPAS1 to suppress its expression, and downregulated EPAS1 resulted in SLC3A2 suppression. Knockdown of EPAS1 and SLC3A2 in HA-VSMCs inhibited the integrin signal pathway molecules’ activity, and cell proliferation, but promoted cell apoptosis. For the first time, it was shown that HCMV-miR-US33-5p suppressed proliferation and promoted HA-VSMC apoptosis by targeting EPAS1/SLC3A2.

HCMV is a double-stranded DNA virus. The positive rate of adult seroantibodies in the world is more than 60–90% [39]. Several studies have suggested that abnormally elevated VSMC apoptosis is correlated with the pathogenesis of AAD [18–20]. Evidence has shown that HCMV can induce apoptosis of several cell types, such as human astrocytes [40], myeloid progenitor cells [41], and neural stem/progenitor cells [42, 43], although HCMV is known to block apoptosis in macrophages and cancer cells [44, 45]. A previous study reported that HCMV can infect VSMCs and affect expression of genes related to cellular lipid metabolism (46). HCMV infection in rat VSMCs induced proinflammatory response through an IκB kinase-related pathway [47]. In the current study, HCMV infection in HA-VSMCs inhibited cell proliferation and promoted cell apoptosis, which may be related to the pathogenesis of AAD. Additionally, the roles of HCMV-encoded miRNAs in cell apoptosis have been described. Liang et al. indicated that HCMV-miR-UL112-3p suppresses the glioblastoma cell apoptosis by targeting and downregulating tumor suppressor candidate 3 [48]. HCMV-miR-US25-1 deteriorates oxidized low-density lipoprotein-induced apoptosis in endothelial cells, promoting atherosclerosis development, as revealed by Fan et al. [49]. Our previous study showed that patients with AAD had significant high HCMV-miR-US33-5p level. In the current study, it was proved that the effects of HCMV infection in HA-VSMCs were mediated by HCMV-miR-US33-5p, and that HCMV-miR-US33-5p mimics elicited the same effects as HCMV infection.

The molecular mechanisms for how HCMV-miR-US33-5p inhibited cell proliferation and promoted cell apoptosis were then investigated. By RNA sequencing and GO-KEGG analysis, it was found that multiple signaling pathways, including integrin signaling, were affected by HCMV-miR-US33-5p mimics in VSMCs. Integrin signaling has been found to be significantly downregulated in dissected aorta tissues of patients with AAD [50]. The ability of integrins to regulate apoptosis is likely due to activation of phosphatidylinositol 3 kinases (PI3K), AKT, or MAPK/ERK [51, 52]. Integrin is known to regulate Rac activity [53]. FAK is a crucial mediator of integrin signaling. Upon activation, FAK associates with either Src or PI3K, which phosphorylated downstream effectors, such as CAS and AKT[54]. The results indicated that HCMV significantly inhibited phosphorylation levels of FAK, AKT, and CAS, and suppressed GTP-Rac through miR-US33-5p level upregulation (Additional file 1: Fig. S1), and that HCMV-miR-US33-5p mimics elicited the same effects as HCMV infection. These findings indicated that the HCMV-miR-US33-5p/integrin signaling axis plays an important role in regulating HA-VSMC apoptosis and may improve our understanding of the pathogenesis of AAD. However, multiple signaling pathways other than integrin signaling are regulated by Rac, FAK, AKT, and CAS. Further studies are needed to explore the implication of these signalings in this process. EPAS1, a hypoxia-inducible transcription factor [26, 27], and SLC3A2, a chaperone protein [28, 29], play important roles in a variety of biological processes, including cell proliferation and apoptosis. Here, EPAS1 and SLC3A2 were identified as genes downregulated by HCMV-miR-US33-5p using RNA sequencing. It was shown that HCMV-miR-US33-5p bound to 3′-UTR of EPAS1 to inhibit its expression, and that EPAS1 bound to the SLC3A2 promoter. Data also demonstrated that EPAS1 silencing significantly reduced SLC3A2 expression. More importantly, it was also proved that silencing EPAS1 or SLC3A2 in HA-VSMCs inhibited cell proliferation and promoted cell apoptosis, consistent with previous reports. Additionally, the activity of key molecules of the integrin signal pathway was also reduced in HA-VSMCs with downregulated EPAS1 or SLC3A2 expression. These data reveal a new role for EPAS1 and SLC3A2 in HA-VSMCs.

The current study has some limitations. More cell lines should be used in the future to test whether the phenomena observed in this study are cell-line specific. Further, in vivo studies using animal models will provide more relevant data. In spite of its shortcomings, this study reveals a new mechanism underlying AAD and may provide a new potential treatment for AAD.

## Conclusions

**Fig. 7:**
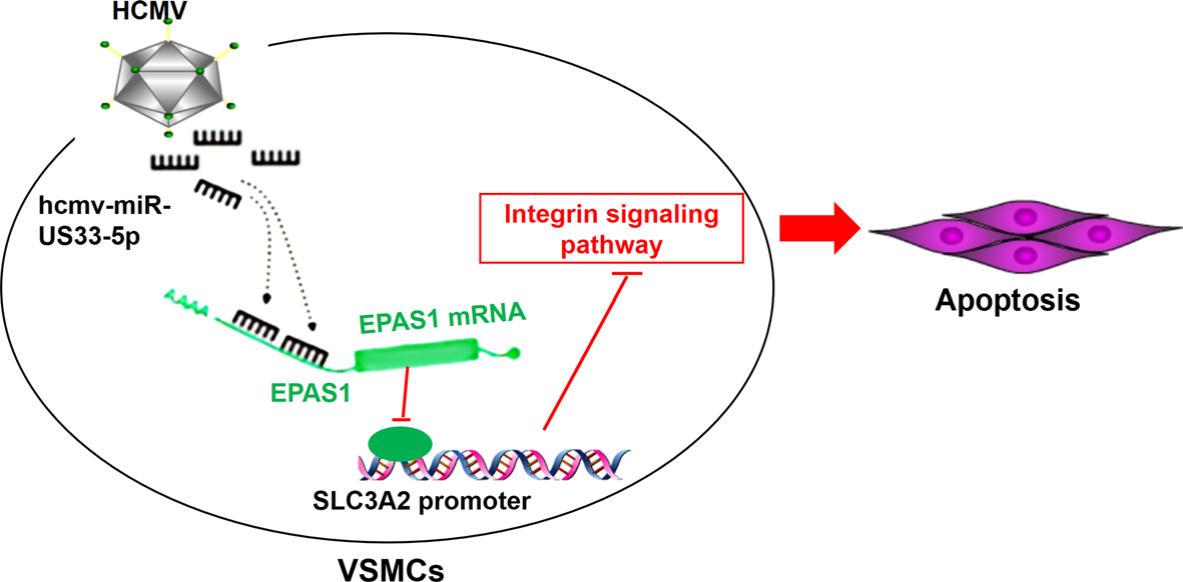
Schematic diagram of signaling pathways involved in HCMV-miR-US33-5p promotion of HA-VSMC apoptosis

This study indicated that HCMV-miR-US33-5p suppressed proliferation but promoted apoptosis of HA-VSMCs, and integrin signaling pathway and EPAS1/SLC3A2 may be involved in this process (Fig. 7). The findings highlighted the importance of HCMV-miR-US33-5p/EPAS1/SCL3A2, which might be helpful in developing new treatment for AAD.

## Supplementary Information

**Additional file 1: Figure S1.** HCMV inhibits key factors of integrin signaling pathway. Western blot analysis of p-FAK (A), p-AKT (B), pY20 CAS (C), and GTP-Rac (D) in HA-VSMCs. Western blot was performed three times; representative images are presented. Numbers above lanes indicate the relative protein level, normalized relative to control samples

**Additional file 2: Table S1.** Upregulated genes in HA-VSMCs transfected with HCMV-miR-US33-5p mimics.

**Additional file 3: Table S2.** Downregulated genes in HA-VSMCs transfected with HCMV-miR-US33-5p mimics.

## Data Availability

All data generated or analyzed during this study are included in this published article.

## Abbreviations

AAD: Acute aortic dissection
HCMV: Human cytomegalovirus
VSMCs: Vascular smooth muscle cells
EPAS1: Endothelial PAS domain protein 1
SLC3A2: Amino acid transporter heavy chain, member 2
HELF: Human embryonic lung fibroblasts
RIP: RNA binding protein immunoprecipitation
CHIP: Chromatin immunoprecipitation
