# Supplementary material for: HCMV-miR-US33-5p promotes apoptosis of aortic vascular smooth muscle cells by targeting EPAS1/SLC3A2 pathway
Source: Cell Mol Biol Lett. 2022 May 20;27:40. doi: 10.1186/s11658-022-00340-w (PMC9123696; doi:10.1186/s11658-022-00340-w)
Supplement: Supplementary file 1 — Additional file 1: Figure S1. HCMV inhibits key factors of integrin signaling pathway. Western blot analysis of p-FAK (A), p-AKT (B), pY20 CAS (C), and GTP-Rac (D) in HA-VSMCs. Western blot was performed three times; representative images are presented. Numbers above lanes indicate the relative protein level, normalized relative to control samples [file 11658_2022_340_MOESM1_ESM.docx]

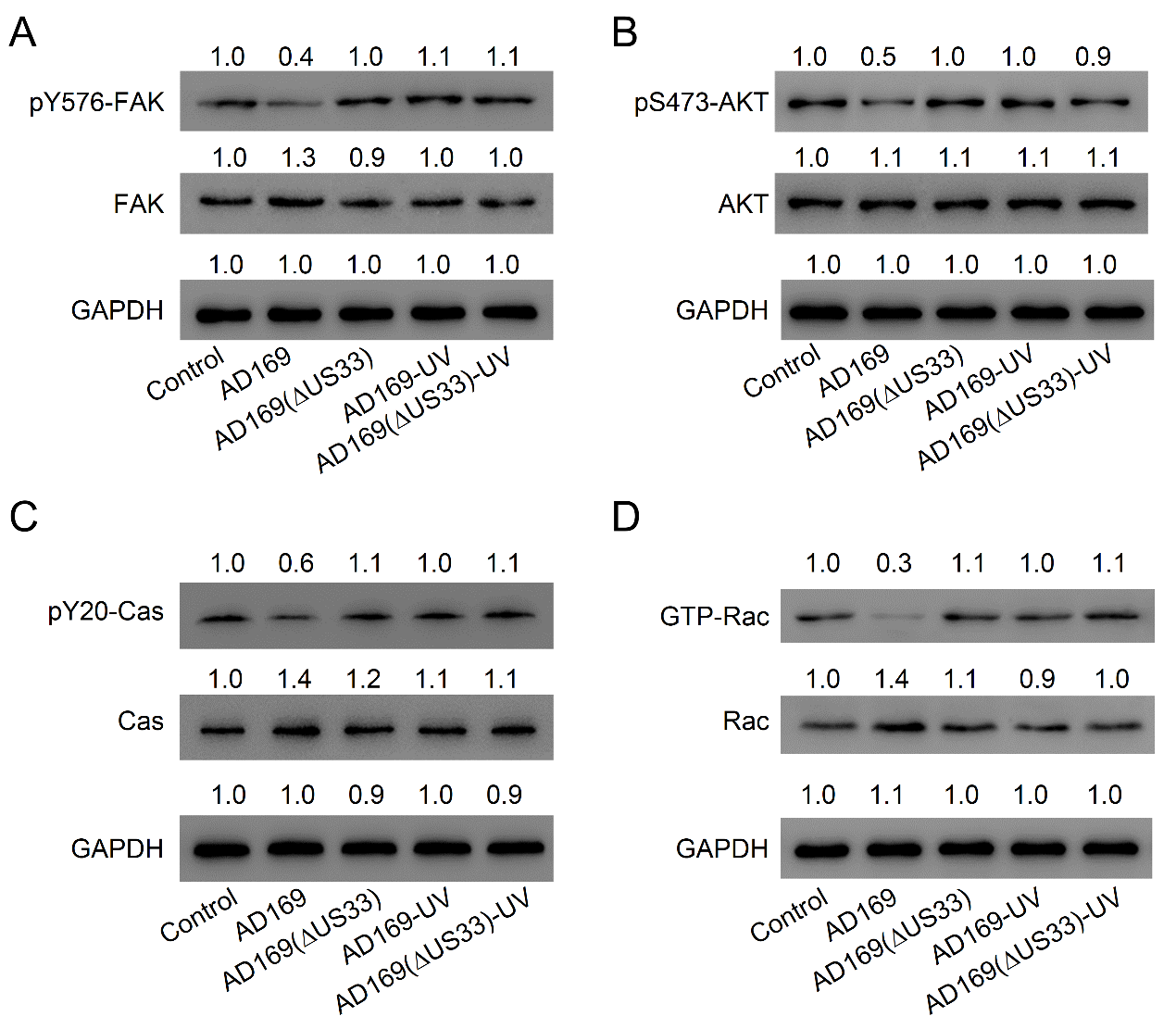


Figure S1. HCMV inhibits key factors of integrin signaling pathway. Western blot analysis of p-FAK (A), p-AKT (B), pY20 CAS (C), and GTP-Rac (D) in HA-VSMCs. Western blot was performed three times and representative images are presented. Western blot was performed three times and representative images were presented. The numbers above the lanes represent the relative protein level, which was normalized relative to the control samples.
